# Porcine vocal fold elasticity evaluation using Brillouin spectroscopy

**DOI:** 10.1117/1.JBO.28.8.087002

**Published:** 2023-08-08

**Authors:** Vsevolod Cheburkanov, Ethan Keene, Jason Pipal, Michael Johns, Brian E. Applegate, Vladislav V. Yakovlev

**Affiliations:** a Texas A&M University, Department of Biomedical Engineering, College Station, Texas, United States; b Tarleton State University, Department of Physics, Stephenville, Texas, United States; c University of Southern California, Caruso Department of Otolaryngology–Head and Neck Surgery, Los Angeles, California, United States; d University of Southern California, Department of Biomedical Engineering, Los Angeles, California, United States

**Keywords:** Brillouin, larynx, *ex vivo*, confocal imaging

## Abstract

**Significance:**

The vocal folds are critically important structures within the larynx which serve the essential functions of supporting the airway, preventing aspiration, and phonation. The vocal fold mucosa has a unique multilayered architecture whose layers have discrete viscoelastic properties facilitating sound production. Perturbations in these properties lead to voice loss. Currently, vocal fold pliability is inferred clinically using laryngeal videostroboscopy and no tools are available for *in vivo* objective assessment.

**Aim:**

The main objective of the present study is to evaluate viability of Brillouin microspectroscopy for differentiating vocal folds’ mechanical properties against surrounding tissues.

**Approach:**

We used Brillouin microspectroscopy as an emerging optical imaging modality capable of providing information about local viscoelastic properties of tissues in noninvasive and remote manner.

**Results:**

Brillouin measurements of the porcine larynx vocal folds were performed. Elasticity-driven Brillouin spectral shifts were recorded and analyzed. Elastic properties, as assessed by Brillouin spectroscopy, strongly correlate with those acquired using classical elasticity measurements.

**Conclusions:**

These results demonstrate the feasibility of Brillouin spectroscopy for vocal fold imaging. With more extensive research, this technique may provide noninvasive objective assessment of vocal fold mucosal pliability toward objective diagnoses and more targeted treatments.

## 1 Introduction

**Fig. 1:**
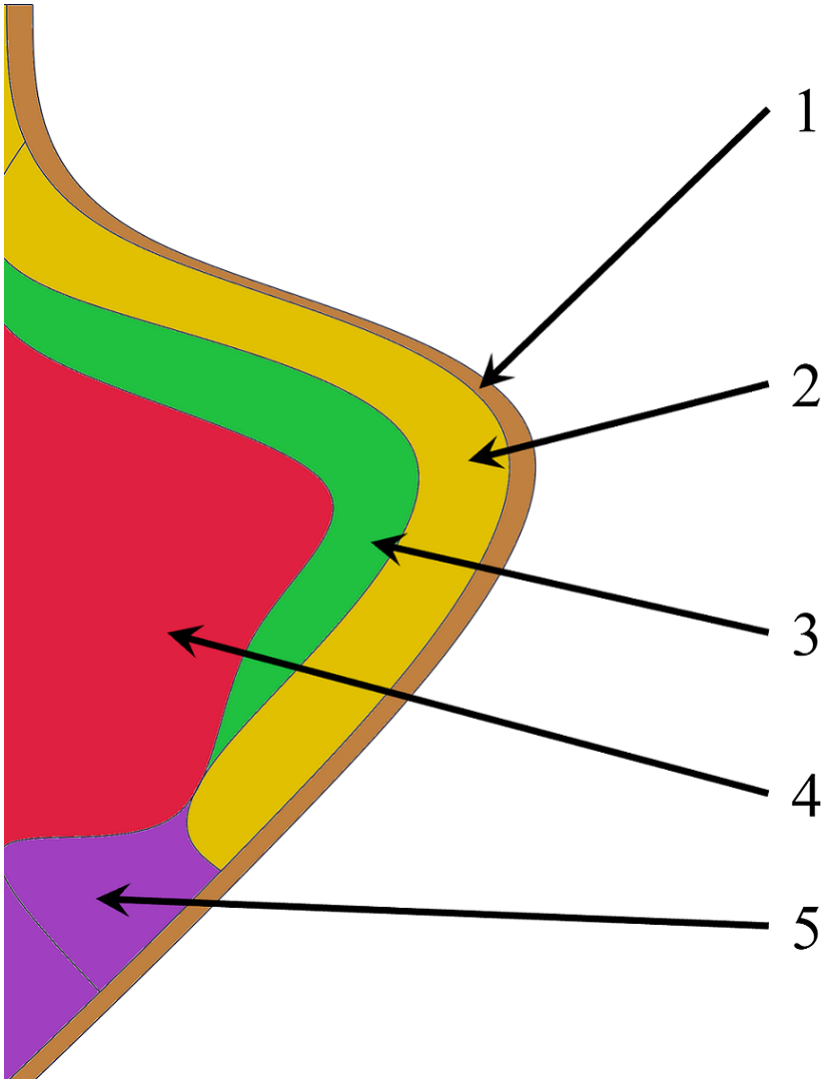
Porcine vocal fold cross-section and tissue layers. 1 – epithelium, 2 – superficial layer (lamina propria), 3 – intermediate layer, 4 – vocalis muscle, and 5 – deep layer.

The larynx serves as a complex valve within the upper aerodigestive tract.^1^ It brokers vital tasks, such as breathing, swallowing, and vocalizing. The larynx contains two vocal folds that drive these primary functions. The vocal folds have a unique multilayered architecture^2^ whose layers have discrete viscoelastic properties favorable for vocalization (Fig. 1).

The vocalis muscle is covered with elastic conus (deep layer), which is composed of dense fibrous tissue, consisting of elastic fibers and collagen. These fibers and collagen are interwoven with vocalis muscle, forming a bond between the two morphologically different layers. The superficial layer is connected to the deep layer loosely through intermediate layer and can move differently from the elastic conus. The superficial layer and epithelium together form the vocal fold mucosa.^3^

The porcine larynx contains two types of vocal folds, similarly to humans; however, unlike the human model, they cannot be definitely addressed as “true” vocal folds and “false” vocal folds due to lack of information regarding which vocal folds play the key role in phonation.^4^ Vocalization is produced via mechanical oscillations of the superficial layers of the true vocal folds induced by subglottal air pressure from controlled exhalation during precisely coordinated vocal fold adduction.

According to previously published research,^5^ lamina propria thickness is around 500  μm and epithelium, which consists of multiple layers of cells,^6^ ∼20 to 100  μm depending on epithelial cell size ranging from 10 to 25  μm.^6^^,^^7^

The vocal folds are susceptible to pathological change, which can alter their mechanical properties resulting in loss of vocal capabilities.^3^ Regardless of the severity of these changes, dysphonia requires accurate diagnosis and treatment. Presently, the larynx and vocal folds are clinically assessed using two-dimensional awake diagnostic laryngoscopy to identify potential pathology aided by videostroboscopy^8^^,^^9^ to infer elements of vocal fold vibration and pliability. Laryngeal tissue morphology and mechanical properties cannot be objectively evaluated with this technique but are rather inferred from improper oscillations of vocal folds.^3^

As a gold standard, definitive diagnosis of laryngeal and vocal fold anomalies requires tissue biopsy for pathological diagnosis that can lead to unintended adverse effects on voice. We propose application of Brillouin microspectroscopy as a viable and non-invasive tool to improve noninvasive diagnostic capabilities for assessing dysphonia and the vocal folds’ mechanical and morphological properties.

Structural evaluation of vocal folds and their mechanical properties can be achieved with the help of more conventional imaging modalities, such as X-ray computed tomography (CT), magnetic resonance imaging (MRI), and ultrasound (US).^10^ While these techniques may provide accurate assessment of anatomical features^11^^,^^12^ and can quantify mechanical properties of vocal folds, they have several substantial drawbacks. CT imaging utilizes X-ray radiation and cannot be used for frequent measurements. MRI is limited in terms of both spatial resolution and the ability to retrieve complex mechanical properties of multilayered voice box tissues. The spatial resolution of US is insufficient for interrogating larynx’ multilayered structure. Optical coherence elastography,^13^ which is based on the acquisition of evolving optical coherence tomography images under external load, often lacks the desired spatial resolution and ability to characterize tissue’s viscous properties.

Brillouin microspectroscopy is an emerging imaging technique built around evaluation of Brillouin scattering.^14^ It can potentially provide quantitative measures of vocal fold mechanical properties^15^^,^^16^ during common endoscopic procedures. Brillouin scattering is a type of inelastic scattering where incident photons interact with phonons within the material^17^^,^^18^ leading to a small change in frequency, which typically does not exceed 20 GHz in biological samples. This frequency change is called the Brillouin frequency shift and its value is related to the material’s high-frequency real elastic modulus, representing stiffness, and imaginary elastic modulus, representing viscosity. According to Vaughan and Randall,^19^ relation between Brillouin shifted line and mechanical properties can be described with the following equation:

$$M(\Delta \nu_{B},\Gamma )=M^{\prime }(\Delta \nu_{B})+jM^{\prime \prime }(\Delta \nu_{B},\Gamma ),$$ (1)

where $\Delta \nu_{B}$ is the Brillouin frequency shift value, and Γ is the Brillouin line full width at half maximum (FWHM) value. The Brillouin frequency shift value is related to the acoustic wave velocity (V) as

$$\Delta \nu_{B}=\pm 2\frac{nV}{\lambda }\;\sin(\frac{\theta }{2}),$$ (2)

where n is the refractive index of the sample, λ is the incident electro-magnetic (EM) radiation wavelength, and θ is the angle between incident and scattering directions. From this equation, the largest observed shift occurs when θ=π, which is known as backscattering geometry. Substituting Eq. (2) into both real and imaginary parts of Eq. (1) results in Eqs. (3) and (4), respectively. Real part of longitudinal modulus is defined by the following equation:

$$M^{\prime }={\left(\frac{\Delta \nu_{B}\lambda }{2n}\right)}^{2}\rho ,$$ (3)

where ρ is material’s mass density. This describes the elastic modulus as proportional to Brillouin frequency shift value squared.

Complex part of the high-frequency longitudinal modulus is defined by the following equation:

$$M^{\prime \prime }=\Delta \nu_{B}\Gamma {\left(\frac{\lambda }{2n}\right)}^{2}\rho .$$ (4)

Brillouin spectroscopic evaluation utilizes relations quantified in Eqs. (3) and (4) allowing for non-destructive label-free determination of viscoelastic properties of various materials. In the past decade, Brillouin spectroscopy has been applied to biological systems numerous times due to its inherent non-invasive and non-destructive nature. Multiple applications in the biological field were aimed at determining mechanical characteristics of different tissue types, such as superficial cancer tissues,^20^ neurons,^21^^,^^22^ eye,^19^^,^^23^^–^^26^ brain,^27^ vasculature,^28^ and bones.^29^

It was reported previously that porcine larynx shares structural similarities with human larynx, making it a suitable model for phonation pathology research.^30^ In this pilot study, we seek to differentiate the pliability of various porcine laryngeal subsites using Brillouin spectroscopy as a step toward potentially using this technology in the clinic. Successful evaluation and differentiation of porcine vocal fold mechanics will pave way to pathophysiology *ex vivo* studies of human larynx in the future.

Previously published research was focused on evaluating porcine larynx structures using cyclic force application and analysis of elongation using a scientific grade ergometer.^31^ Here, we hypothesized that data acquired in previous studies would correlate with data acquired using a Brillouin spectrometer as described herein.

To this end, we acquired a batch of porcine larynxes, tuned the system to allow for highly scattering sample imaging, and devised the experiment to remotely measure the elastic properties of excised porcine laryngeal structures using a confocal Brillouin spectrometer.

**Fig. 2:**
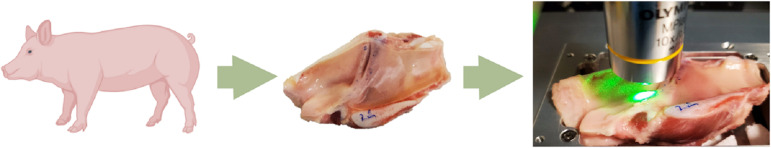
Experimental flowchart. Created with BioRender.^32^

The flowchart in Fig. 2 provides a graphic description of the experimental flow.

As shown in Fig. 2, regions of interest were highlighted on the exposed larynx lining with a thin marker to ease the process of tracking and later statistical analysis.

We targeted superior vocal fold (SVF) and inferior vocal fold (IVF) of the porcine larynx with the expectation of their mechanical properties being significantly different from surrounding tissue. The surrounding supraglottal wall (SGW) was also evaluated as comparator non-vocal fold tissue.

## 2 Materials and Methods

### 2.1 Setup Description and Acquisition Settings

**Fig. 3:**
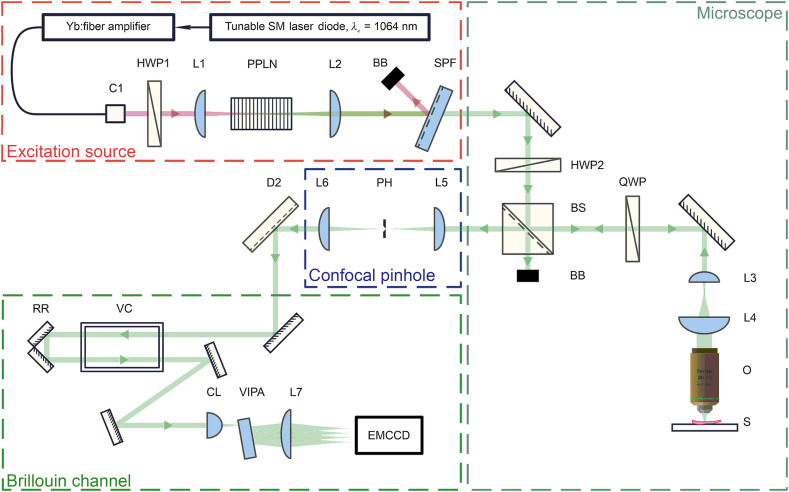
Brillouin confocal microspectrometer layout. (BB: beam block; C1: 1064 nm FC/APC fiber collimator; CL: cylindrical lens; D2: dichroic mirror; EMCCD: electron-multiplying charge coupled device detector; HWP1: 1064 nm half-wave plate; HWP2: 532 nm half-wave plate; L1, L2, and L7: plano-convex lens; L3, L4, L5, and L6: cemented achromatic doublets; O: objective; BS: broadband visible range polarizing beamsplitter cube; PH: precision pinhole; PPLN: periodically poled second harmonic generating lithium niobate crystal; QWP: 532 nm quarter-wave plate; RR: hollow-roof prism retroreflector; S: sample; SPF: 750 nm short-pass filter; VC: Iodine reference vapor cell; and VIPA: virtually image phased array).

Data were acquired in backscattering geometry using a custom-built upright confocal Brillouin microspectrometer,^20^^,^^33^^,^^34^ as shown in Fig. 3.

Brillouin scattering was induced with 532 nm radiation, which was generated as a result of second harmonic generation in a periodically poled lithium niobate (PPLN) (Fig. 3) crystal (MSHG1064-1.0-20, Covesion Ltd.) pumped by amplified 1064 narrowband tunable single longitudinal mode laser diode (Koheras Adjustik and Koheras Boostik HPB, NKT Photonics). Optimal phase matching conditions are achieved by rotating a 1064 nm half-wave plate (HWP1) (Fig. 3) (Thorlabs). Residual 1064 nm pump is separated from the beam by means of a tilted short-pass filter (SPF) with a 750 nm cutoff wavelength and is directed toward a beam block (BB) (Fig. 3). Linearly polarized laser radiation passed through a HWP2 (Fig. 3) (Thorlabs) before entering a broadband polarizing beamsplitter (BS) cube (Fig. 3) (Thorlabs). Rotating the HWP2 allowed us to precisely control the power level at the sample. Excess 532 nm radiation transmitted through the polarization beam splitter (PBS) is diverted toward a BB (Fig. 3). A quarter-wave plate (QWP) (Fig. 3) (Thorlabs) in conjunction with the BS formed a free space circulator, which allowed us to effectively steer light to the sample and from the sample into the acquisition arm. A custom Keplerian beam expander (L3/L4, Fig. 3) was used to improve fill factor of microscope objective (O) lens (Fig. 3) entrance pupil. Microscope objective (CFI Plan Fluor 20X, 0.5 NA Nikon) was used for simultaneous delivery of 5±0.3  mW of 532 nm radiation to the sample (S) (Fig. 3) and collection of scattered photons.

Brillouin microspectrometer axial resolution did not exceed 6  μm. It was measured using laser beam knife edging technique at the sample plane. Spot size at the same time did not exceed 4  μm. These laser beam parameters were chosen for optimal combination of interrogated volume size, laser power density, spatial resolution, and low noise from backscattered photons.

Collected light was guided toward a confocal pinhole assembly. Radiation was focused into a precision pinhole (PH) (Fig. 3) (National aperture) with an achromatic doublet (L5, Fig. 3) and recollimated with lens (L6, Fig. 3). Superior lateral and axial resolution of ∼0.6 and 2 mm, respectively, was achieved through selection of sub-airy unit pinhole.^35^

Spatially filtered radiation was transmitted through a heated iodine vapor cell (VC) (Fig. 3) (Thorlabs).^36^ This provided full suppression of the elastically scattered spectral line after setting the VC temperature to 70°C, using a double pass beam propagation geometry and fine-tuning 1064 nm LD output wavelength to match the strongest absorption band of iodine. Optimal absorption was achieved at 531.9363 nm, which, according to molecular iodine vapor absorption spectra atlas,^37^ corresponds to line 638.

Following excitation suppression, the beam was directed to a custom-built single stage virtually image phased array (VIPA) (Fig. 3) spectrometer. Free spectral range of the utilized VIPA (OP-6721-3371-2, Light Machinery Inc.) was 29.98 GHz ($1\;{\mathrm{cm}}^{-1}$). Coatings on the VIPA were optimized for 532 nm applications. Cylindrical lens (CL1) (Fig. 3) (Thorlabs) and long focal distance imaging plano-convex lens (L7, Fig. 3) (Thorlabs) were chosen to allow for five orders of the spectral pattern to be imaged on the sensor. A water cooled electron-multiplying charge coupled device (EMCCD) detector camera (Andor Newton 970P, Oxford Instruments) was used to detect inelastically scattered photons at high speed. Signal to noise ratio (SNR) exceeded 3.0 for transparent homogeneous samples (acetone). The camera sensor was operating in full vertical binning mode with each acquisition lasting 100 ms. Data shifting frequency of 50 kHz was chosen to minimize readout noise. During time intervals when the sensor was processing data or waiting for command, two mechanical iris-style shutters (Thorlabs) were used to block any radiation on the sensor active area and the sample.

Each imaged area was sampled 100 times in a 10×10 pattern with 1  μm step between the interrogated points to allow for data averaging and sample preservation. In other words, we acquired signal from a $10\times 10\;{\mu m}^{2}$ field of view, with multiple pixel overlap.

Axial distance between the objective and microscope stage was fixed, meaning that topography of the tissue was not compensated for in the duration of the scan. However, sampling size, imaging depth, 6  μm axial resolution, spectral filtration features, and structure of porcine larynx allow us to disregard effects of sample topography fluctuations.

Each interrogated region was targeted individually to achieve highest imaging depth, which was located between 25 and 35  μm. Low axial resolution of 6  μm and large spot size of 4  μm in diameter, allowed for elasticity averaging within a large volume of tissue. Molecular iodine absorption cell, heated to 70°C typically contaminated acquired spectra with iodine absorption lines if signal was acquired from sample boundary. This meant that to achieve clean Brillouin scattering spectra, we had to image the sample no shallower than 3  μm. And porcine larynx lamina propria typically displays thickness values of ∼500  μm. With these factors in mind, we assumed that fluctuations in topography of the sample are locally inconsequential.

To find imaging position, we observed spectral data in real time and slowly lowered the objective lens to the sample. Once iodine spectral features were visible and signal was maximized, we recorded objective position since laser waist was on the boundary of the sample. We then proceeded to lower the objective down, imaging tissues deeper inside the larynx, until iodine features’ counts were smaller than Brillouin frequency shift counts. These conditions were typically met at the depth between 25 and 35  μm. These values were recorded via measurement of displacement of a 3D microscope piezo stage with respect to the sample’s surface. Once position was found, lateral scanning algorithm was initiated.

Imaging plane axial movement in case of an irregular surface is accounted for during signal analysis. In case iodine spectral features are prominent and saturate Brillouin spectral lines from larynx, these points are dropped from statistical analysis due to them being outside 25 to 35  μm region. The same approach is employed when dealing with weak signal from beyond 35  μm of depth.

### 2.2 Sample Preparation

**Fig. 4:**
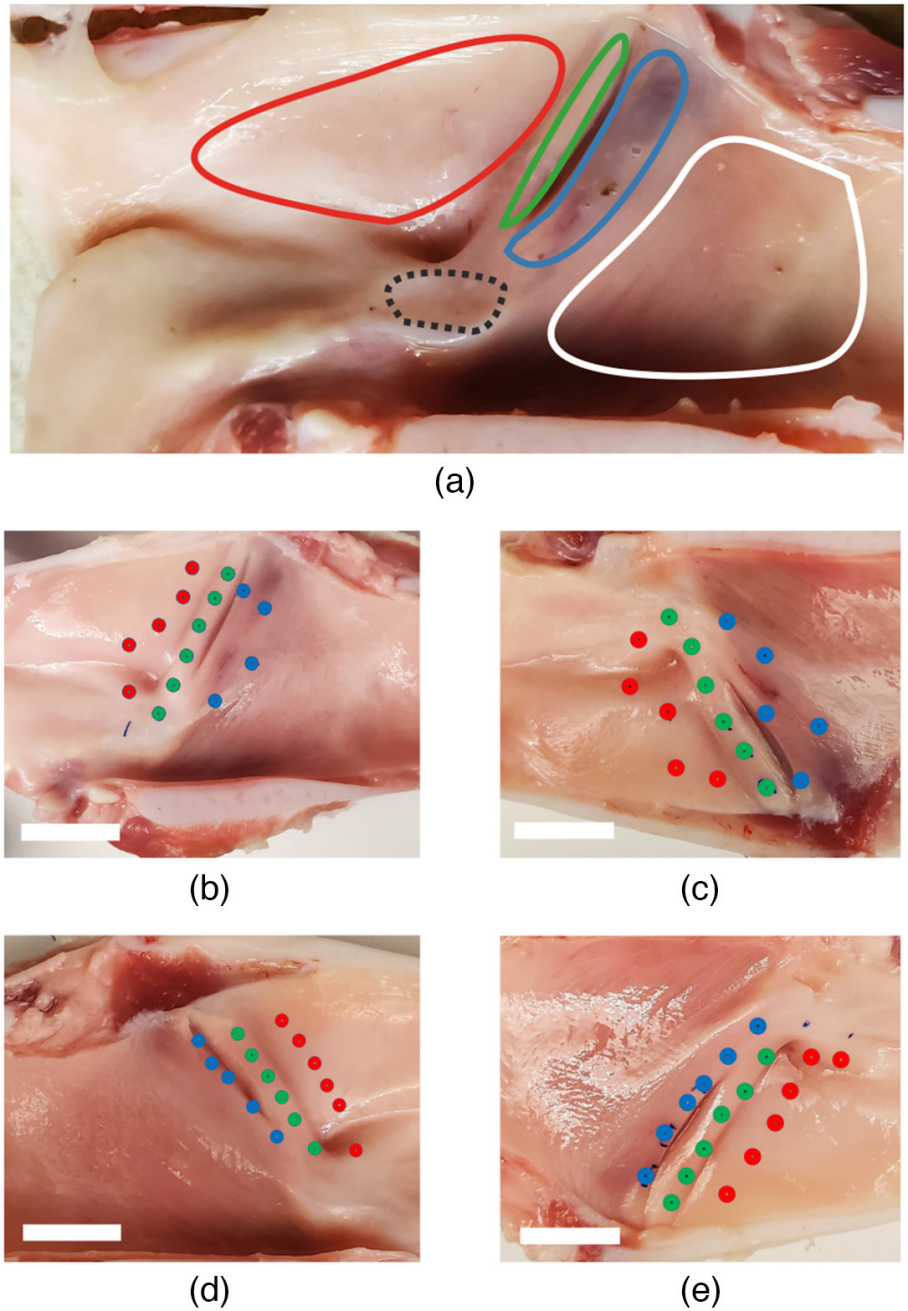
(a) Lateral half after a vertical midline section of a pig larynx sample. Highlighted regions are: SGW (red), SVF (green), IVF (blue), subglottal Wall (white), and arytenoid (black). (b)–(e) Samples prepared for interrogation with each region selected are labeled consecutively from top to bottom. White bar shows a 15 mm scale.

A post-mortem fresh pig larynx model was used in this study. This model is a viable substitution of human larynx for mechanical evaluation.^2^^,^^4^ Samples were sourced from 3-month-old healthy animals and purchased from Animal Biotech Industries, delivered overnight, and stored in the refrigerator at 4°C with administration of light coat of PBS to preserve superficial tissue layers. A midline vertical section was created through the larynx to allow for successful imaging of its various constituents Fig. 4(a).

Accumulation of a dataset sufficient for statistical analysis, multiple separate porcine larynx samples, which are shown in Figs. 4(b)–4(e), were prepared. Three functional sites per sample were chosen for interrogation: the tissue below the true vocal folds (SGW), the true vocal folds (SVF), and the false vocal folds (IVF) [Figs. 4(b)–4(e)]. Each of the sites was sampled multiple times, and the array of dots of respective color in Figs. 4(b)–4(e) highlights each point of interest within the functional site. Interrogated points were randomly selected within the region of interest to provide a sparse coverage of the interrogated area.

### 2.3 Signal Processing Algorithm

**Fig. 5:**
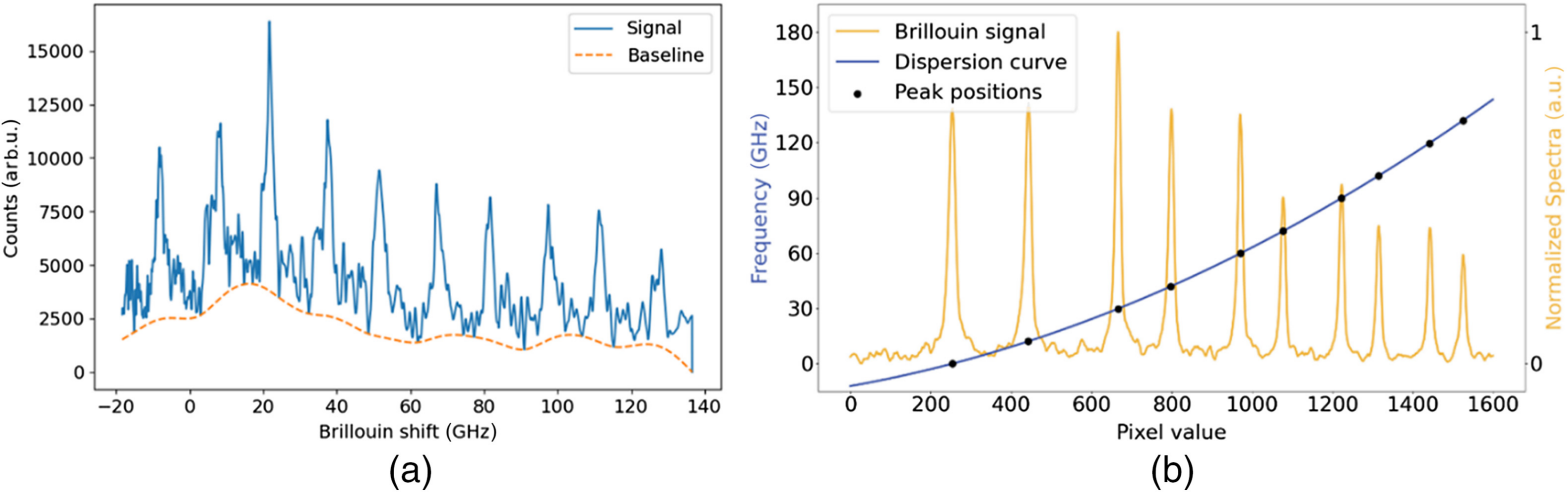
(a) Retrieved porcine larynx Brillouin scattering signal (solid line) and baseline curve (dashed line) and (b) retrieved acetone Brillouin scattering spectra, registered from acetone at 20°C (orange line), VIPA dispersion curve (blue line).

Acquired spectra were stored as 1D arrays before signal processing. The first step was median filtration with a three-element kernel, which was meant to suppress impulse noise (e.g., cosmic rays). This was followed by baseline subtraction. Baseline curve was calculated via the use of an asymmetric least-squares fit algorithm [Fig. 5(a)].

The processed signal was then converted from pixel domain to the GHz domain via approximation of VIPA dispersion curve using a clear and homogeneous sample as a reference (acetone sealed in a quartz cuvette). Stokes and anti-Stokes peak pairs retrieved from the acetone sample along with the fit peak maxima positions and VIPA dispersion curve are shown in Fig. 5(b).

According to previously published results,^36^ the dispersion curve of VIPA spectrometers can be approximated by a third order polynomial for the goal of converting pixel results to GHz with high accuracy and low computational load. Each registered peak in the spectra was fitted with a single Lorentzian curve, with the cumulative spectra being a superposition of each fit. Peak positions were recorded and used for least squares fit to a third order polynomial.

At the time of the measurements on the porcine vocal folds, spectrometer performance was characterized in a form of systematic error measurement. It was approximated from 100 data points acquired from acetone at 20°C. The histogram and Gaussian fit are shown in Fig. 4.

**Fig. 6:**
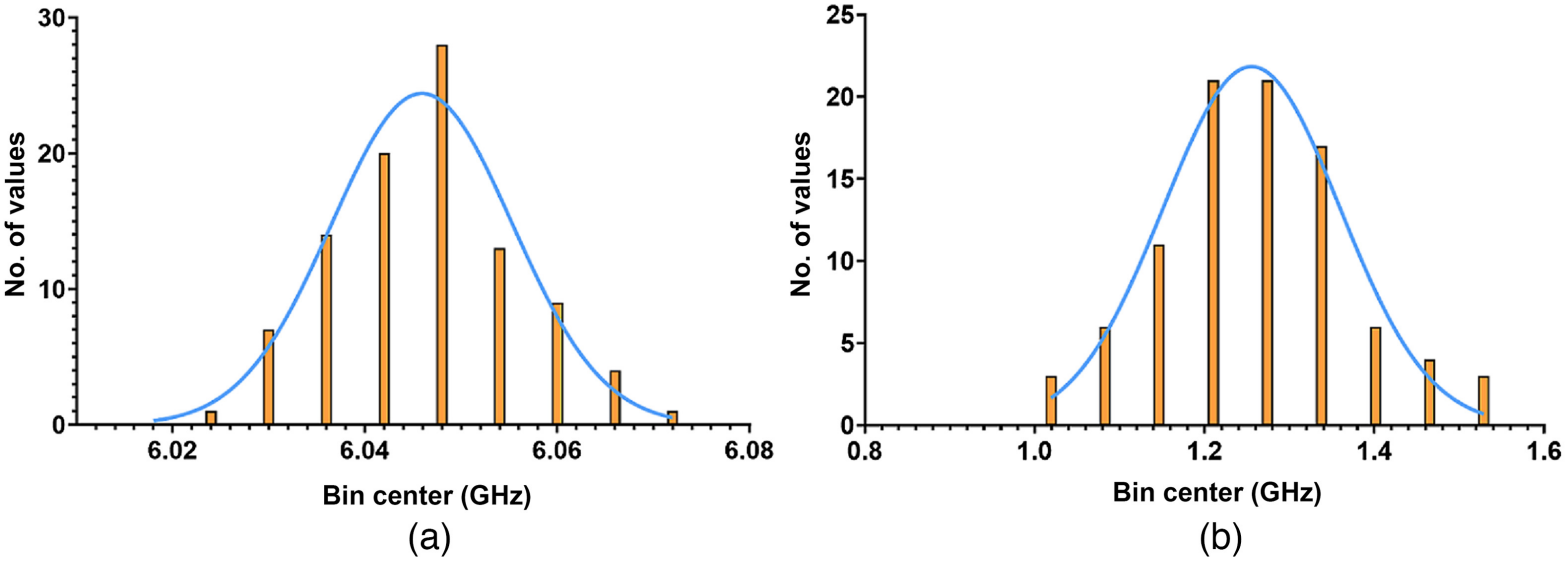
(a) Brillouin frequency shift value distribution for acetone at 20°C and (b) Brillouin line FWHM value distribution for acetone at 20°C. Orange bar plot represents number of values. Blue line represents Gaussian fit.

As seen in Fig. 6, value of Brillouin frequency shift value is 6.046±0.0094  GHz, and mean Brillouin line FWHM value is 1.262±0.11  GHz. All the described here Brillouin measurements were performed at a fixed room temperature of 20°C.

## 3 Results and Discussion

Compiled results for studied laryngeal samples are presented in the figures listed below. Three laryngeal regions that were interrogated for their elastic properties are shown in Figs. 4(b)–4(e). Specific points of interrogation are highlighted as: blue dots for the IVF region, green dots for the SVF region, and red dots for the SGW region. Multiple points were measured from each region, labeled consecutively from the top as indicated by the arrows.

To account for tissue heterogeneity, digital reduction of system resolution was employed. Statistical analysis of frequency shift and linewidth value data within a sampled region provided raw standard deviation values for each interrogated region. Outliers were filtered out through a combination of median filtration, statistical analysis, and smoothing of 2D value map within a sampled subregion.

**Fig. 7:**
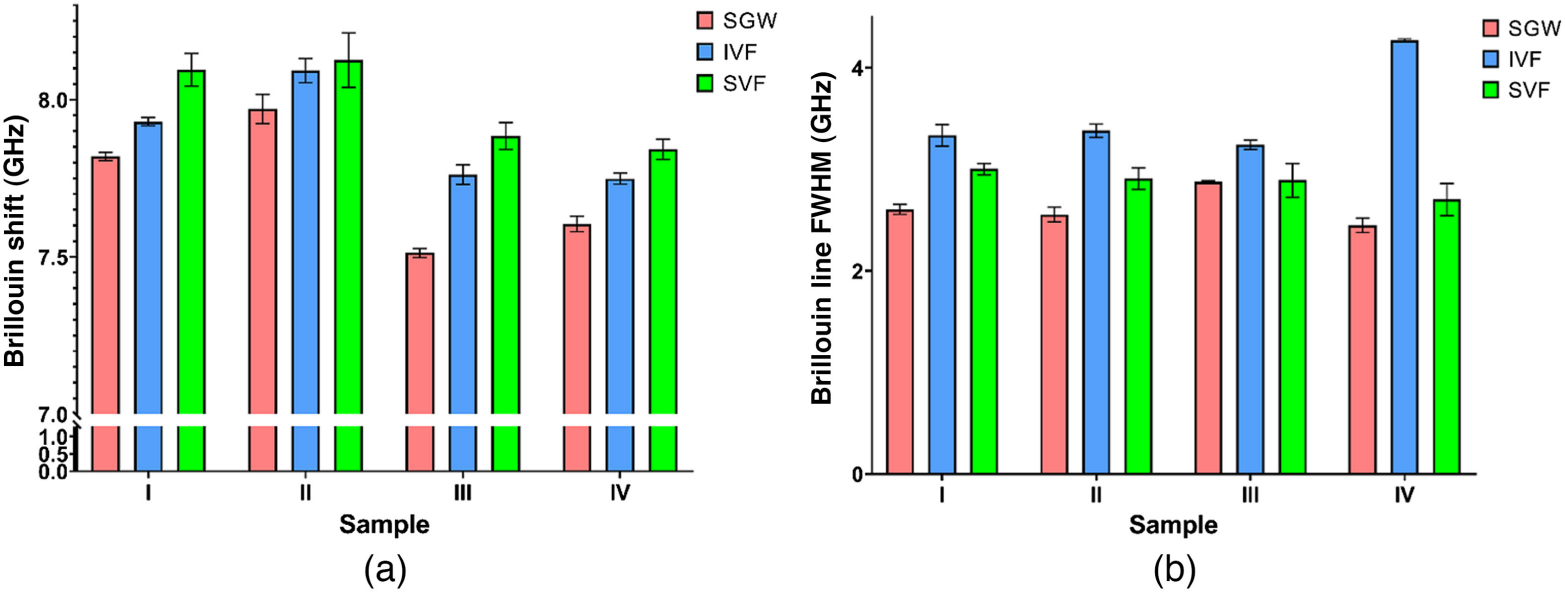
(a) Larynx sample cumulative Brillouin frequency shift value statistics and (b) Brillouin line FWHM statistics.

Total accumulated data of mean retrieved values of Brillouin frequency shift and Brillouin line width are presented as bar plots in Fig. 7.

Brillouin frequency shift and Brillouin line FWHM data follow the same trend among all interrogated samples. Compiled data are shown in Table 1. The statistical significance of data from different regions was evaluated using Mann-Whitney tests.

**Table 1:** Brillouin frequency shift and Brillouin line FWHM data.

Sample	SGW		IVF		SVF	
	Shift (GHz)	FWHM (GHz)	Shift (GHz)	FWHM (GHz)	Shift (GHz)	FWHM (GHz)
I	7.82 ± 0.01	3.00 ± 0.06	7.93 ± 0.01	3.33 ± 0.11	8.10 ± 0.05	2.60 ± 0.05
II	7.97 ± 0.05	2.55 ± 0.07	8.0 ± 0.04	3.38 ± 0.06	8.12 ± 0.09	2.91 ± 0.10
III	7.51 ± 0.014	2.88 ± 0.01	7.76 ± 0.03	3.24 ± 0.04	7.88 ± 0.04	2.89 ± 0.17
IV	7.60 ± 0.02	2.45 ± 0.07	7.75 ± 0.02	4.27 ± 0.02	7.84 ± 0.03	2.70 ± 0.16

All the interrogated regions retrieved Brillouin frequency shift value distributions that were significantly different (p<0.0001) from one another, with one exception: the difference in Brillouin frequency shift value of sample #2 SGW region was statistically insignificant compared to frequency shift value statistics of SVF and IVF region of the same sample.

**Fig. 8:**
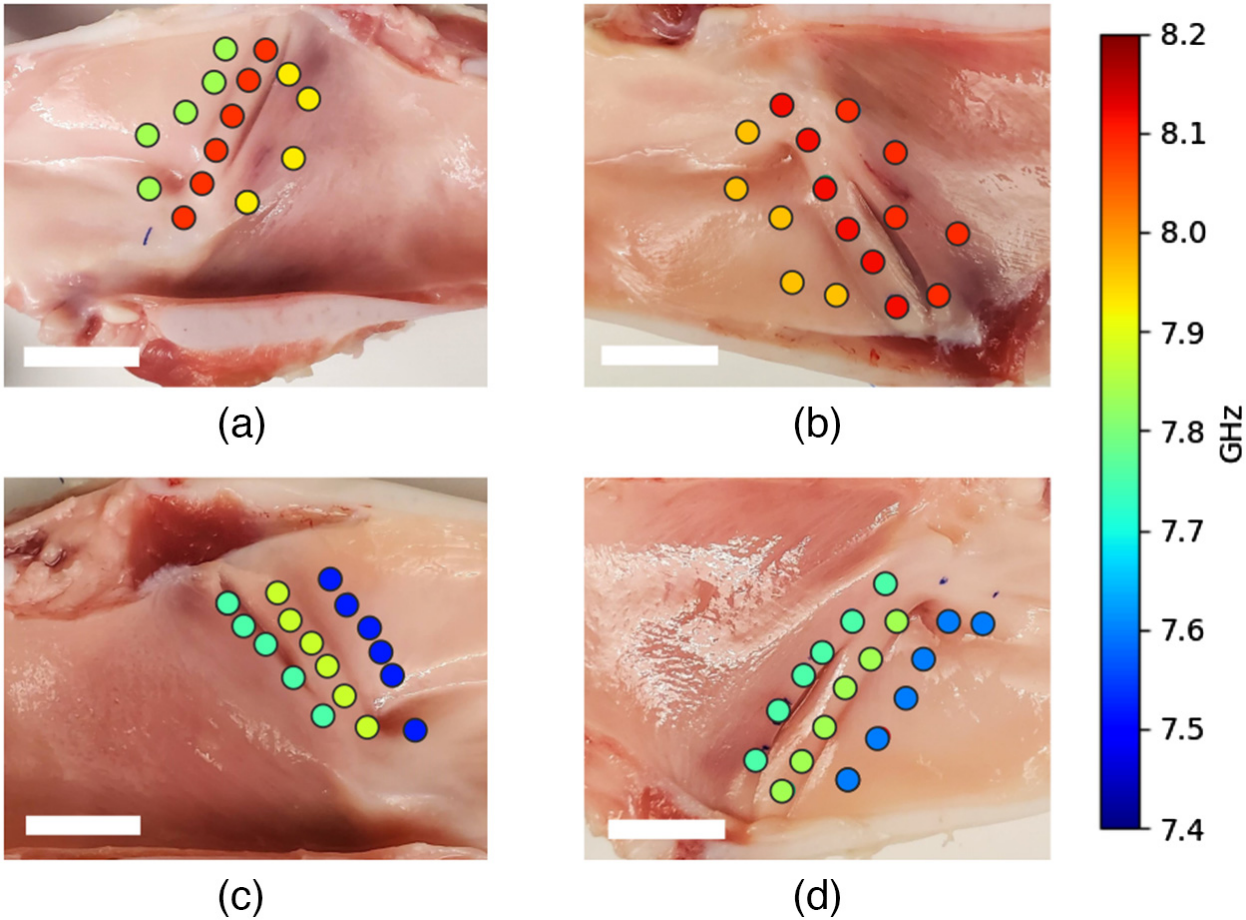
Brillouin frequency shift values overlapped on larynx sample images. (a)–(d) Samples I through IV, respectively. White bar shows a 15 mm scale.

**Fig. 9:**
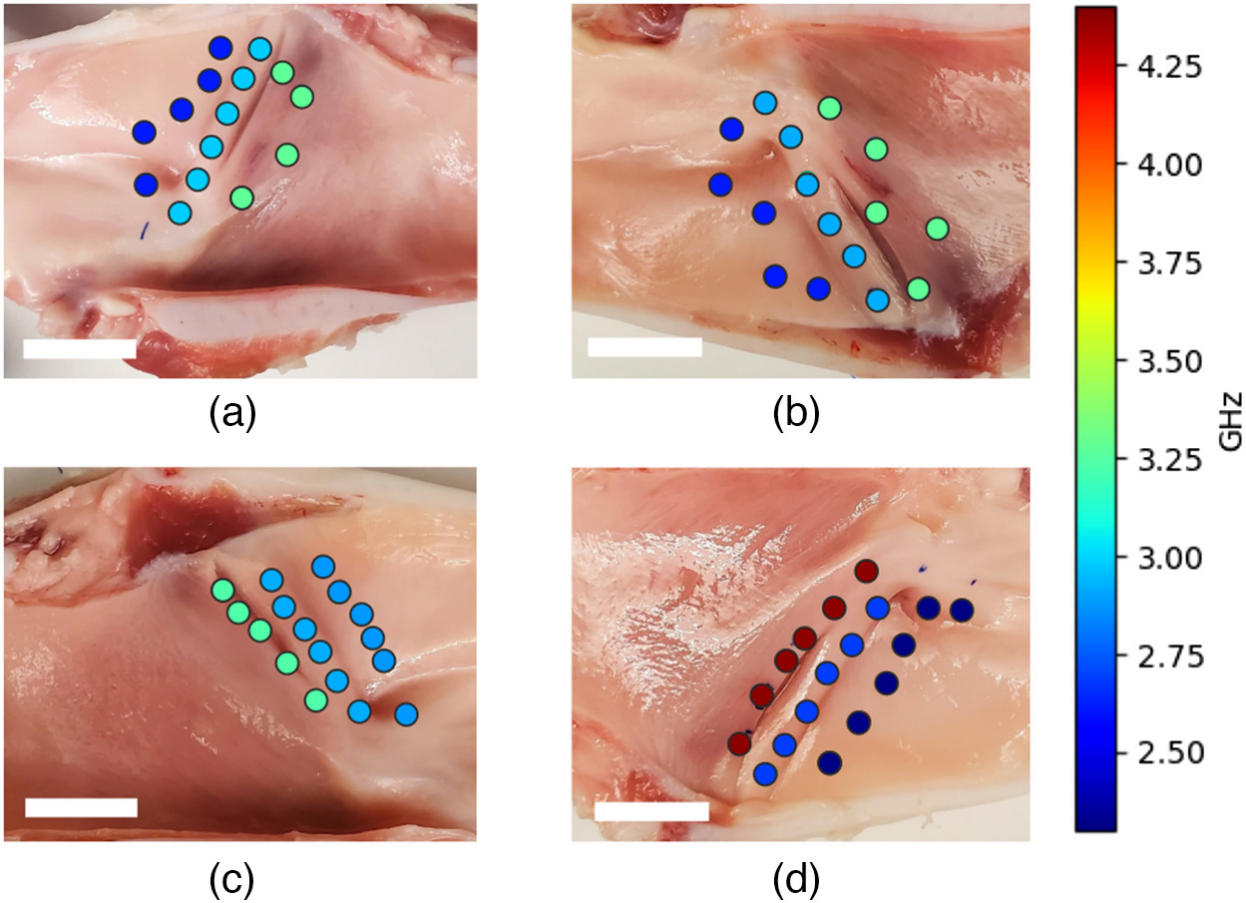
Brillouin line FWHM values overlapped on larynx sample images. (a)–(d) Samples I through IV, respectively. White bar shows a 15 mm scale.

Brillouin line frequency shift and FWHM values retrieved from interrogated regions are visualized in Figs. 8 and 9. Number of points shown in Figs. 8 and 9 is not consistent between the regions, due to some of the points being excluded from statistical analysis.

Data shown in Table 1 show the following trend.

• SVF region in all four samples displays the highest Brillouin frequency shift value, and SGW displays the lowest Brillouin frequency shift value, with IVF displaying median Brillouin frequency shift value.
• Brillouin line FWHM values in IVF regions of the larynx are observed to be greater than those of SVF and SGW regions.

These trends can be explained by the respective cellular structure of the laryngeal tissues and the cellular response to extracellular matrix (ECM) stiffness,^38^ which has also been validated using Brillouin microspectroscopy imaging.^39^ Taking into consideration that our level of penetration lies between 25 and 35  μm, we conclude that we are interrogating the epithelial cells of laryngeal lining.

The SGW region displayed the lowest Brillouin frequency shift values and lowest Brillouin line FWHM values, suggesting that this region has the highest elasticity and lowest viscosity. According to histological analysis of porcine larynx,^7^ this region has the largest thickness of epithelium and underlying cartilage substrate. Low values of retrieved Brillouin shifts may be explained by gradual decrease of stiffness going from cartilage superficially toward outermost layers. And due to the large thickness of the epithelium layer, this mechanical signal from ECM wanes to the softest outer layers of SGW that we observed in our study.

Vocal folds, on the other hand, have been shown to have thin epithelium, attached to collagen-rich lamina propria.^5^ With the known imaging depth, we can interpret high Brillouin frequency shift values the following way. Epithelium cells are exposed to high-stiffness^40^ collagen-rich ECM, which drive cell stiffness. Such comparative Brillouin frequency shift difference may occur due to the difference in epithelium thickness or lamina propria morphology and stiffness.

Previously published results were acquired using classical evaluation techniques that utilized the entire volume of excised vocal fold. Since, from the volumetric point of view, lamina propria makes up for the larger part of the total volume of vocal folds, we can infer that mechanical properties of vocal folds are driven by it. In case of our study, this means that Brillouin frequency shift difference occurs due to high lamina propria stiffness and different morphology between IVF and SVF. This also implies that due to good arrangement with previously published data, our system is indirectly evaluating lamina propria stiffness.

## 4 Conclusions

The ability of Brillouin microspectroscopy to differentiate between laryngeal substructures was successfully demonstrated for the first time. The use of porcine larynx allowed for the comparison of Brillouin frequency shift values between SVF, IVF, and SGW regions of the larynx. The Brillouin frequency shift values were statistically significantly different, with SGW tissue displaying the lowest Brillouin frequency shift value, and SVF displaying the highest, in agreement with previously published results using traditional mechanical testing.

While these results clearly demonstrate that porcine vocal folds can be differentiated against surrounding tissue using Brillouin frequency shift values, further study is needed. The promise of Brillouin microspectroscopy for characterizing the vocal fold mechanical properties is that it is noninvasive and can potentially be done using common endoscopic approaches for imaging the vocal folds in the office setting. If changes in the mechanical properties that correlate with vocal fold pathology can be observed with Brillouin microspectroscopy, this approach could become a powerful diagnostic tool for the laryngologist.

## Code, Data, and Materials Availability

All data in support of the findings of this paper are available within the article.
